# Autologe Knorpelzelltransplantation mit Knochenaufbau zur Behandlung osteochondraler Defekte am Knie: Behandlung osteochondraler Defekte der Femurkondylen mittels autologer Spongiosazylinder aus dem Beckenkamm kombiniert mit matrixgestützter autologer Chondrozytentransplantation

**DOI:** 10.1007/s00064-021-00751-6

**Published:** 2021-11-29

**Authors:** Christoph Stotter, Stefan Nehrer, Thomas Klestil, Philippe Reuter

**Affiliations:** 1 Abteilung für Orthopädie und Traumatologie, Landesklinikum Baden-Mödling, Sr. M. Restituta-Gasse 12, 2340 Mödling, Österreich; 2 Zentrum für Regenerative Medizin, Donau-Universität Krems, Dr.-Karl-Dorrek-Str. 30, 3500 Krems an der Donau, Österreich

**Keywords:** Knorpelschaden, Knie, Osteochondrale Rekonstruktion, Autologe Spongiosaplastik, Subchondraler Knochen, Cartilage defect, Knee, Osteochondral reconstruction, Cancellous bone grafting, Subchondral bone

## Abstract

**Operationsziel:**

Offene Therapie osteochondraler Läsionen des Kniegelenks zur vollständigen Auffüllung knöcherner Defekte und Wiederherstellung der Gelenkfläche.

**Indikationen:**

Fokale, symptomatische osteochondrale Defekte des Kniegelenks ab einer Knochendefekttiefe von ≥ 5 mm und Größe von ≥ 1,5 cm^2^.

**Kontraindikationen:**

Arthrose (> KL Grad 2), „kissing lesions“ (ICRS > Grad 2), Alter > 50 Jahre, unbehandelte Bandinstabilitäten oder Beinachsenfehstellungen zum Nachteil des betroffenen Gelenkkompartiments, fehlendes Defektcontainment, entzündliche Gelenkerkrankungen.

**Operationstechnik:**

Zweizeitiger Eingriff: Ersteingriff (arthroskopische Knorpelprobenentnahme): Defektevaluierung, Entnahme von Knorpelstücken für die Chondrozytenkultivierung, bei Bedarf Behandlung von Begleitpathologien. Zweiteingriff (offene Defektbehandlung): Arthrotomie, Präparation des knöchernen Defekts, Auffüllung mit autologen Spongiosazylindern aus dem Beckenkamm, Knorpeldefektpräparation (kann größer als knöcherner Defekt sein) und matrixgestützte autologe Chondrozytentransplantation.

**Weiterbehandlung:**

Ersteingriff: frühfunktionelle Nachbehandlung mit schmerzorientierter Vollbelastung je nach Begleiteingriffen. Zweiteingriff: keine Drainage, funktionelle Kniegelenkorthese in Streckstellung für 1 Woche, danach schrittweise Freigabe der Flexion, Teilbelastung für 6 Wochen, Motorschiene (CPM) ab dem 1. postoperativen Tag.

**Ergebnisse:**

Seit 2018 sind 8 Patienten (mittleres Alter 29,4 Jahre, Spanne 18 bis 36) mit der beschriebenen Technik behandelt worden. Alle Patienten konnten nach durchschnittlich 12 Monaten nachuntersucht werden. Der Gesamt-KOOS (Knee injury and Osteoarthritis Outcome Score) verbesserte sich im Mittel von 45,8 auf 81,3, und die postoperativen radiologischen Kontrollen zeigten die Einheilung der Spongiosazylinder bei allen Patienten. Der MOCART (Magnetic Resonance Observation of Cartilage Repair Tissue) Score ergab 80,4 Punkte.

## Abstract (en)

**Objective:**

Open treatment of osteochondral defects of the knee to fully restore the bony defect and the articular surface.

**Indications:**

Focal, symptomatic osteochondral lesions of the knee with a depth of ≥ 5 mm and a surface of ≥ 1.5 cm^2^.

**Contraindications:**

Osteoarthritis, kissing lesions, age > 50 years, untreated ligament instability or relevant malalignment, insufficient containment, inflammatory joint disease.

**Surgical technique:**

Two-step procedure: Step 1 (arthroscopy): evaluation of the defect, harvesting of cartilage tissue for chondrocyte cultivation, if needed treatment of concomitant pathologies. Step 2 (open surgery): arthrotomy, preparation of the defect, transfer of iliac crest cancellous bone plugs to the defect, preparation of the cartilage defect (can exceed the size of the bony defect), matrix-guided autologous chondrocyte transplantation.

**Postoperative management:**

Step 1: therapy follows the needs of concomitant procedures, with early full weight bearing as tolerated. Step 2: no suction drain, knee brace in extension for week 1, followed by stepwise increase of flexion, partial weight bearing for 6 weeks, continuous passive motion starting on postoperative day 1.

**Results:**

Since 2018, 8 patients (mean age 29.4 years) have been treated with the described technique. All patients were available for follow-up examination after an average of 12 months. The KOOS (Knee injury and Osteoarthritis Outcome Score) improved from 45.8 to 81.3 and the postoperative X‑rays showed bony healing of the cancellous bone plugs in all patients. The MOCART (Magnetic Resonance Observation of Cartilage Repair Tissue) score reached 80.4 points.

## Vorbemerkungen

Ausgedehnte, symptomatische osteochondrale Defekte am Kniegelenk bedürfen einer operativen Versorgung [1]. Da oft junge, aktive Patienten betroffen sind, ist ein gelenkerhaltender Eingriff mit dem Ziel der Schmerzreduktion und Wiederherstellung der Gelenkfunktion anzustreben. Zusätzlich kann durch eine Rekonstruktion der osteochondralen Einheit die frühe Entstehung der Osteoarthrose verhindert werden [2].

Zur knöchernen Defektauffüllung, begleitend zu einem knorpelrekonstruktiven Eingriff, stehen abhängig von der Größe des Defektes die Auffüllung mit autologen oder homologen Spongiosachips [1, 3], der autologe Knochenblock aus dem Beckenkamm [1] oder die Auffüllung mittels autologer Spongiosazylinder zur Verfügung [4, 5]. Zusätzlich stellt die Verwendung von osteochondralen Allografts eine Behandlungsoption dar, die aufgrund der eingeschränkten Verfügbarkeit in Europa zurzeit eine untergeordnete Rolle spielt [6].

Die knöcherne Auffüllung mittels Spongiosachips in Kombination mit einer Knorpelzelltransplantation ist der Behandlung von kleineren Defekten (Defekttiefe < 10 mm) vorbehalten [1], weist bei dieser Technik jedoch eine niedrige Primärstabilität auf. Bei kleinen Defekten, die mittels eines einzigen osteochondralen Zylinders behandelt werden können, stellt die autologe osteochondrale Transplantation (AOT) eine gute Behandlungsoption dar. Größere Defekte eignen sich hingegen nicht für eine AOT, da bei der Verwendung von 2 oder mehreren Knorpel-Knochen-Zylindern im Sinne einer „Mosaikplastik“ die Ergebnisse aufgrund einer inkompletten Defektdeckung und erhöhten Entnahmemorbidität schlechter sind [7].

Die Defektbehandlung mit autologem kortikospongiösem Block aus dem Beckenkamm und begleitender Knorpeltransplantation eignet sich für große und tiefe osteochondrale Defekte sowohl als Primär- als auch als Revisionseingriff [1]. Insbesondere auch notchnahe Defekte können durch die Krümmung des bikortikalen Beckenkammspans rekonstruiert werden. Für diese Technik konnte auch in biomechanischen Studien gezeigt werden, dass die maximale Belastbarkeit des Spans vergleichbar ist mit jener von entknorpelten Femurkondylen [8].

Nach rein ossärer Defektauffüllung kommen zur Rekonstruktion des artikulären Knorpels und der osteochondralen Einheit abhängig von Defektgröße, Lokalisation, Patientenalter und Aktivitätsniveau unterschiedliche knorpelregenerative Verfahren zum Einsatz. Vor allem bei großen Knorpeldefekten am Kniegelenk (≥ 2,5 cm^2^) stellt die autologe Chondrozytentransplantation (ACT) den Goldstandard dar [9] und bietet Vorteile im Vergleich zu matrixgestützten, zellfreien Verfahren (z. B. AMIC®, Geistlich Pharma AG, Wolhusen, Schweiz). Die ACT kann hierbei sowohl im matrixgestützten Verfahren als auch in Form von Sphäroiden aus matrixassoziierten Chondrozyten erfolgen [10, 11].

## Operationsprinzip und -ziel

Offene Auffüllung eines osteochondralen Defektes an den Femurkondylen mit autologen Spongiosazylindern aus dem Beckenkamm kombiniert mit matrixgestützter autologer Knorpelzelltransplantation.

## Vorteile

- Lückenlose Rekonstruktion des knöchernen Defekts möglich
- Pressfit-Fixierung der Spongiosazylinder mit hoher Primärstabilität ohne zusätzliche Implantate
- Hohes regeneratives Potenzial der Beckenkammspongiosa
- Behandlung von großen Knorpeldefekten mit nur kleinem knöchernem Defekt möglich
- Matrixgestützte Chondrozytenimplantation auch bei großen Knochendefekten durchführbar
- Integration des Knorpelregenerats am Defektrand in den gesunden Knorpelwall
- Keine Entnahmemorbidität im Kniegelenk, verglichen mit AOT

## Nachteile

- Entnahmemorbidität am Beckenkamm
- Technisch anspruchsvolles Verfahren
- Grenzen der Technik bei sehr großen knöchernen Defekten (> 5 cm^2^)
- Zweizeitiges Vorgehen
- Kostenintensives Verfahren durch die Chondrozytentransplantation

## Indikationen

- Fokaler, symptomatischer osteochondraler Defekt am Kniegelenk
- Osteochondritis dissecans mit knöchernem Defekt und Containment
- Revisionseingriff nach knorpelrekonstruktivem Eingriff mit Beteiligung des subchondralen Knochens
- Knochendefekttiefe ≥ 5 mm

## Kontraindikationen

- Unbehandelte Gelenkinstabilitäten oder Beinachsenfehlstellung
- Alter > 50 Jahre (Ergebnisse der isolierten MACT deutlich schlechter)
- Arthrose, fortgeschrittene Knorpelschäden in den restlichen Gelenkabschnitten
- Fehlendes Defektcontainment bei notnahen Defekten
- Kniegelenkinfekt

## Patientenaufklärung

- Allgemeine Risiken: z. B. Infektionen, Thromboembolie, postoperative Schmerzen
- Verletzung des Ramus infrapatellaris des N. saphenus
- Schmerzen an der Entnahmestelle am Beckenkamm, Nachblutung, iatrogene Fraktur, Verletzung des N. cutaneus femoris lateralis, Infektion
- Postoperativer Hämarthros und Notwendigkeit einer Punktion (keine Redondrainage)
- Insuffiziente Regeneratbildung, Delamination, Fusionsstörung oder Hypertrophie des Knorpelregenerats
- Auftreten von subchondralen Ödemen oder Zysten
- Arbeitsunfähigkeit mindestens 8 Wochen
- Langes Nachbehandlungsregime mit Sportrückkehr frühestens 1 Jahr postoperativ

## Operationsvorbereitung

- Allgemeinzustand, Operationsrisiken
- Klinische Untersuchung
- Röntgenaufnahme des Kniegelenks in 2 Ebenen und Patella tangential
- Ganzbeinaufnahme im Stehen
- Computertomographie (CT) zur exakten Vermessung des knöchernen Defekts
- Magnetresonanztomographie (MRT) zur Beurteilung des Knorpeldefekts und Begleitpathologien (Knochenmarködem, Knorpelstatus in anderen Gelenkabschnitten, Menisken)
- Reizfreies Kniegelenk mit annähernd freier Beweglichkeit

## Instrumentarium

### Arthroskopie

- Standard-Knie-Arthroskopieinstrumentarium
- Instrument zur Entnahme von Knorpelgewebe für Knorpelzellzüchtung (ca. 2 × 5mm)

### Offene Defektauffüllung

- Übliches Operationsinstrumentarium für Kniegelenkarthrotomie
- Hohlstanzensystem zur Entnahme und zum Transfer von Knochenzylindern (z. B. Single Use-OATS®; Arthrex, Naples, FL, USA)
- Oszillierende Säge, Meißelset
- Ringküretten, Kugel- oder Birnenfräse zur exakten Defektpräparation
- Material und Instrumentarium zur Knorpelzelltransplantation je nach Hersteller

## Anästhesie und Lagerung

- Allgemein- oder Spinalanästhesie (cave: Beckenkammentnahme)
- Rückenlage
- Anlage einer Oberschenkelblutsperre
- Hohe Seitenstütze am Oberschenkel und Positionierungsblocks in verschiedenen Flexionsgraden, alternativ elektrischer Beinhalter
- Waschen und Abdecken auch an der Entnahmestelle am Beckenkamm
- Perioperative Antibiose

## Operationstechnik

**(**Abb. 1, 2, 3, 4, 5, 6, 7, 8, 9, 10, 11 und 12**)**.

**Figure 1:**
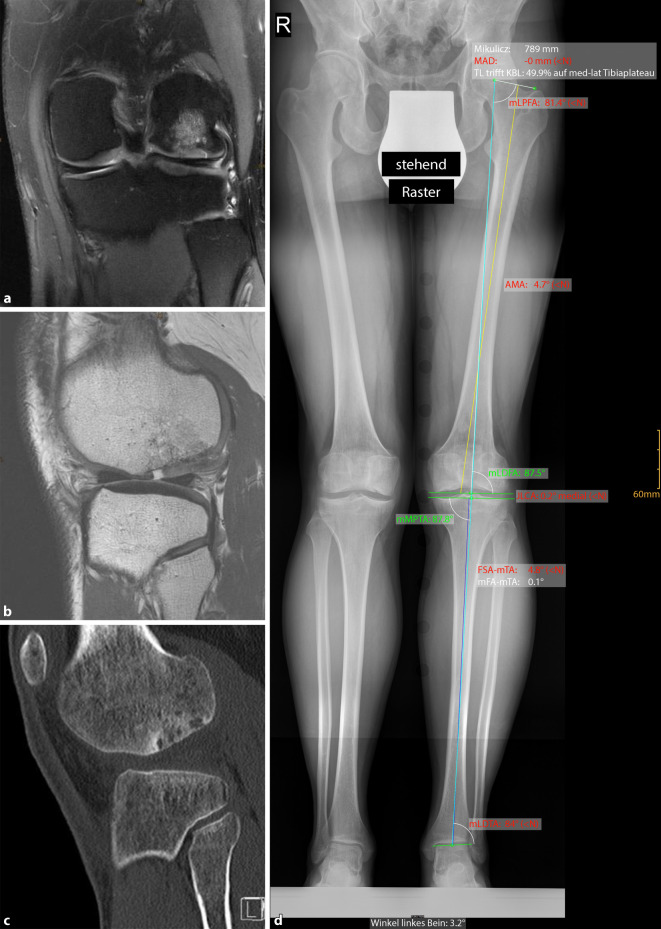

**Figure 2:**
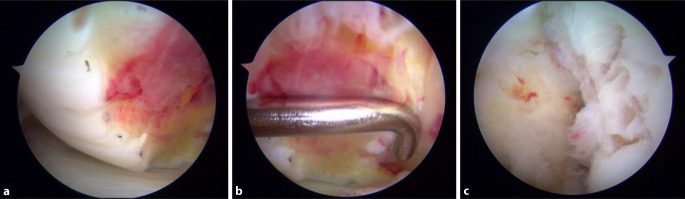

**Figure 3:**
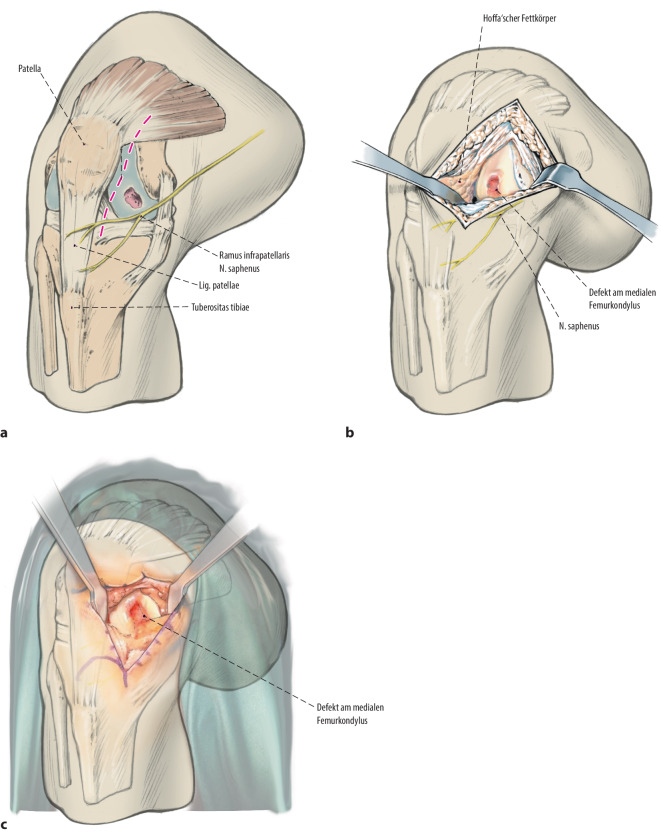

**Figure 4:**
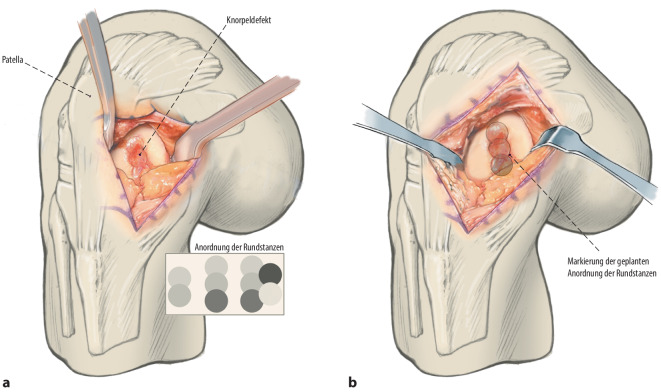

**Figure 5:**
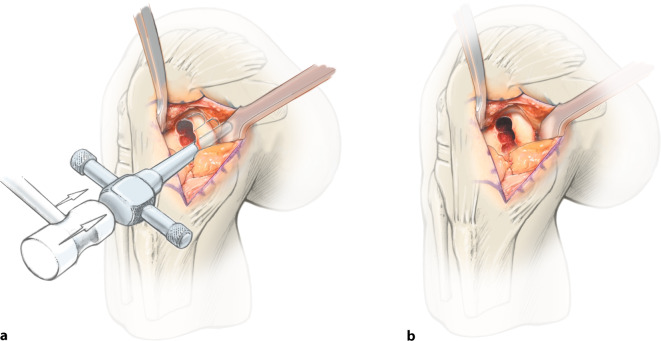

**Figure 6:**
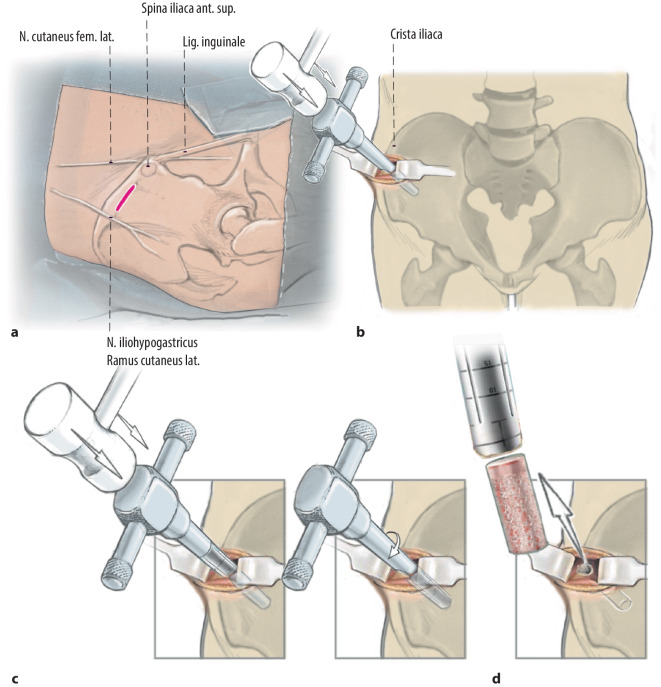

**Figure 7:**
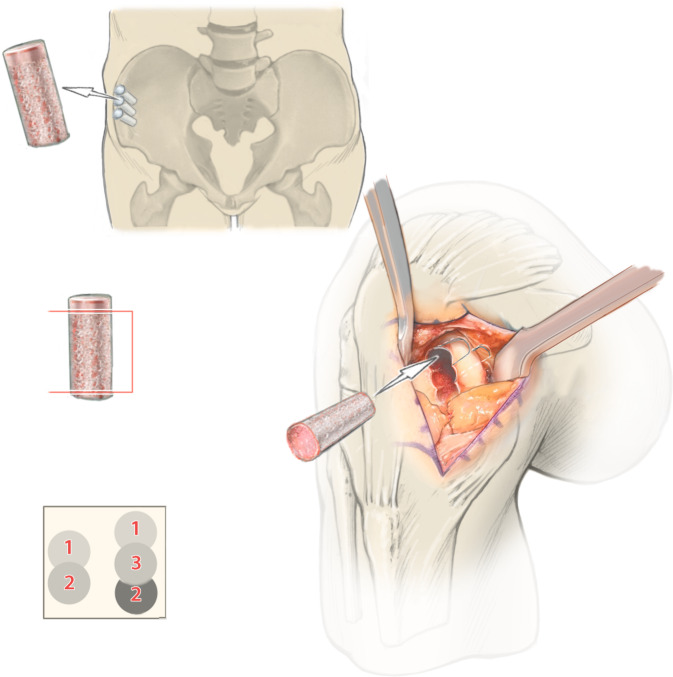

**Figure 8:**
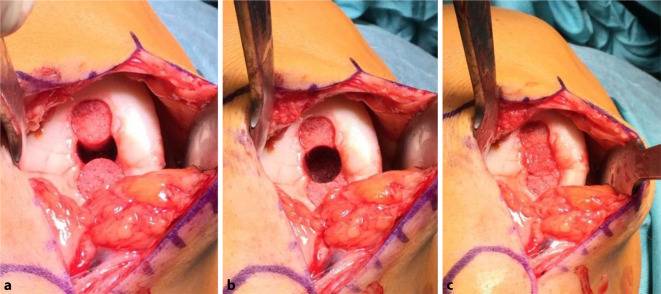

**Figure 9:**
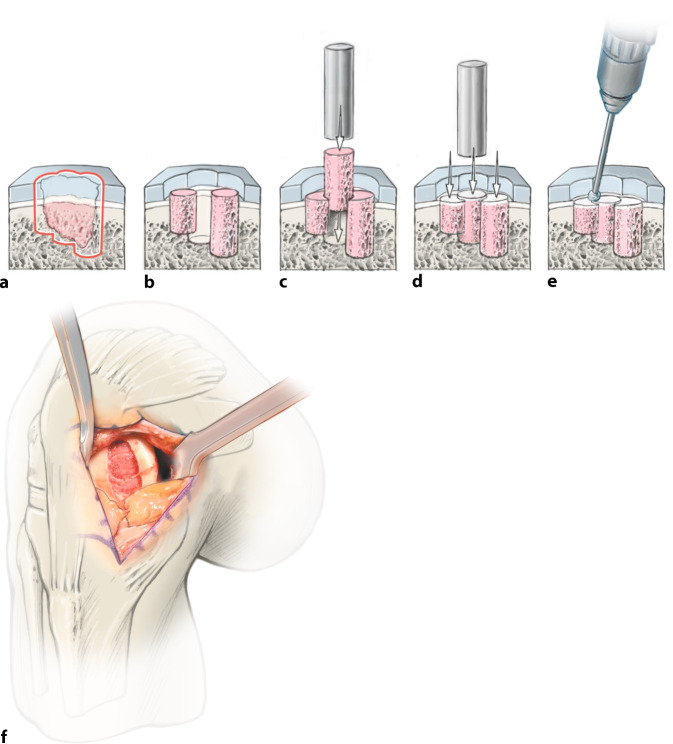

**Figure 10:**
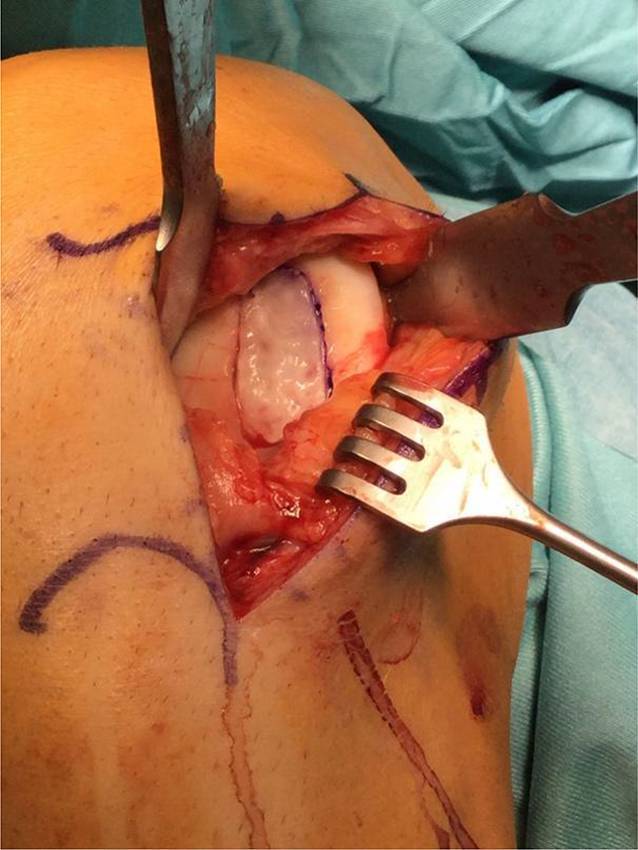

**Figure 11:**
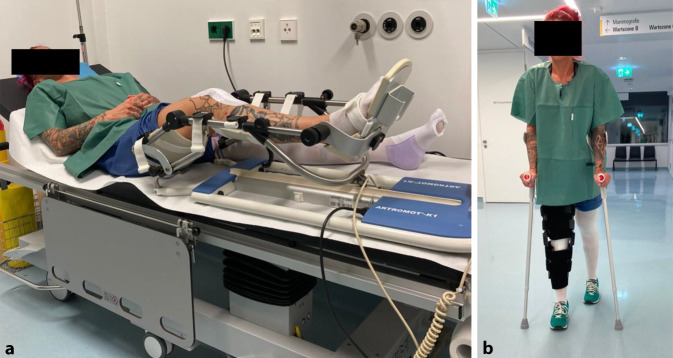

**Figure 12:**
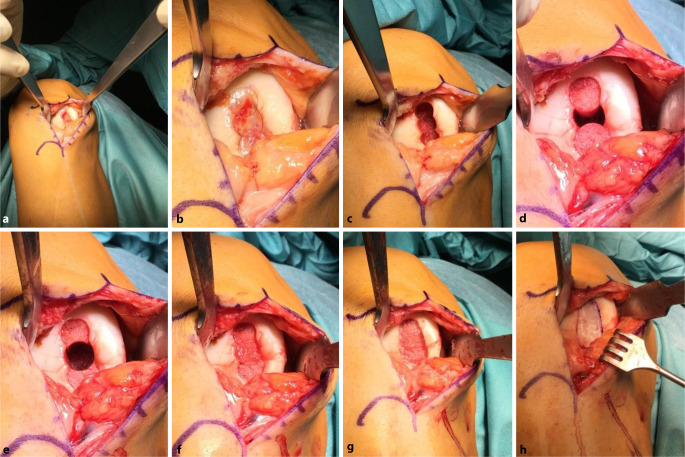

## Besonderheiten der Operationstechnik

In der beschriebenen Technik werden spongiöse Zylinder zur knöchernen Defektauffüllung verwendet und in Press-Fit-Technik verankert. Im Unterschied dazu werden in der von Ochs et al. beschriebenen Technik [4] kortikospongiöse Zylinder verwendet. Die Verwendung von spongiösen Zylindern ermöglicht eine einfache und exakte Modellierung des Knochens. Durch eine überlappende Anordnung der Zylinder wird eine lückenlose Auffüllung des Defektes erreicht. Durch Kompaktierung mit einem Stößel kommt es zu einer Verdichtung v. a. der oberflächlichen Anteile und zur Ausformung einer neuen subchondralen Lamelle. Diese kann im Anschluss mit Kugel- oder Birnenfräse bearbeitet und die konvexe Krümmung der Femurkondylen genau rekonstruiert werden. Danach kann die matrixgestützte Chondrozytentransplantation je nach Hersteller in standardisierter Technik durchgeführt werden.

## Postoperative Behandlung

- Keine intraartikulären Lokalanästhetika, keine Redondrainage
- Anlage eines sterilen Wundverbands und elastokompressive Bandagierung des Beines
- Durch die hohe Primärstabilität der ossären Auffüllung wird die postoperative Behandlung ausschließlich von der MACT bestimmt
- Die Rehabilitation erfolgt unter physiotherapeutischer Anleitung
- Initial Anlage einer Kniegelenkorthese (z. B. medi M.4 X‑lock®, medi GmbH, Bayreuth, Deutschland) in Streckstellung für 1 Woche, danach schrittweise Freigabe der Flexion
- Motorschiene zur passiven Mobilisation täglich ab dem 1. postoperativen Tag 0‑0-30°mit Steigerung der Flexion um jeweils 5° bei Schmerzfreiheit. In der 3. postoperativen Woche sollte eine passive Flexion bis 90° möglich sein, in der 6. postoperativen Woche eine Flexion bis 120°
- Entlastende Mobilisation mit Bodenkontakt für 2 Wochen, danach schrittweise Zunahme der Belastung, ab der 5. postoperativen Woche Belastung mit halbem Körpergewicht und Übergang zur Vollbelastung ab der 7. postoperativen Woche
- Arbeitsausfall mindestens 8 Wochen

## Fehler, Gefahren, Komplikationen

- Insuffiziente Entnahme der Zylinder mit kortikaler Wand – ausreichend großer Hautschnitt und Präparation des Beckenkamms, exakte Ausrichtung der Stanzen
- Auftreten von subchondralen Ödemen oder Zysten – bei Symptomatik evtl. Subchondroplastie
- Delamination des Regeneratknorpels – bei guter Qualität des Regenerats nach Knorpelzelltransplantation und Einheilung der Knochenzylinder – evtl. Refixation mittels Naht
- Komplettversagen – Ausschluss von unbehandelten Komorbiditäten wie Beinachsenfehlstellungen, danach evtl. neuerliche MACT, bei großen Defekten Gelenkersatz (Teiloberflächenersatz, Schlittenprothese, Totalendoprothese)

## Ergebnisse

**Figure 13:**
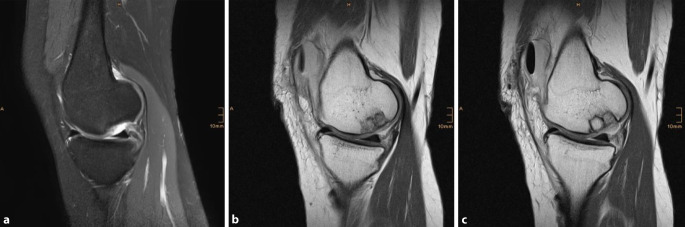

Seit 2018 haben wir 8 osteochondrale Defekte am Kniegelenk bei 8 Patienten (mittleres Alter 29,4 Jahre, Spanne 18 bis 36) mit der beschriebenen Technik behandelt. Die Defekte waren am medialen (6/8) und lateralen (2/8) Femurkondyl lokalisiert. Je nach Defektgröße wurden 1 bis 5 Spongiosazylinder verwendet. Der Nachuntersuchungszeitraum liegt bei durchschnittlich 12 Monaten (4 bis 24 Monate), wobei für 4 Patienten eine MRT-Kontrolle nach zumindest 12 Monaten vorliegt. Die klinischen Scores verbesserten sich bei allen Patienten bei der Follow-up-Untersuchung, verglichen mit präoperativen Werten. Der KOOS Score verbesserte sich im Mittel von präoperativ 45,8 auf 81,3 zum Zeitpunkt der letzten Nachuntersuchung. Die postoperativen radiologischen Kontrollen zeigten die Einheilung der Spongiosazylinder bei allen Patienten. Die verfügbaren postoperativen MRT-Untersuchungen zeigten die stufenlose und vollständige Wiederherstellung der subchondralen Lamelle in korrekter Höhe bei 7/8 Patienten (Abb. 13). Im Beobachtungszeitraum wurde keine Delamination oder Transplantatversagen der MACT beobachtet. Der mittlere MOCART Score lag nach 12 Monaten bei 80,4. Es wurden weder Wundinfektionen, noch thromboembolische Ereignisse oder postoperative Arthrofibrose beobachtet. Die Ergebnisse sind vergleichbar mit jenen der vorher publizierten Techniken.

Zellner et al. berichteten in einem vergleichbaren Patientenkollektiv (Durchschnittsalter 28,2 Jahre, Defektgröße 6,7 cm^2^) eine kontinuierliche Verbesserung des IKDC Scores von präoperativ 42,6 auf 75,3 nach 1 Jahr und 84,3 nach 3 Jahren [1]. Defekte mit ≤ 10 mm Tiefe wurden mittels Spongiosaplastik und Defekte mit ≥ 10 mm Tiefe mittels Beckenkammspan jeweils in Kombination mit MACT behandelt. Der MOCART Score 1 Jahr postoperativ betrug 82,6, wobei insbesondere der subchondrale Knochen eine zufriedenstellende Regeneration zeigte.

Ochs et al. [4] verwendeten zur knöchernen Auffüllung und Rekonstruktion der subchondralen Platte kortikospongiöse Zylinder kombiniert mit MACT. Insgesamt wurden 26 osteochondrale Läsionen (ICRS OCD III und IV) mit einer durchschnittlichen Größe von 5,3 cm^2^ und Defekttiefe von 8,7 mm behandelt. Die Patienten zeigten bei der Follow-up-Untersuchung nach 2 bis 5 Jahren eine signifikante Verbesserung in allen klinischen Scores (Lysholm-Gillquist Score, Cincinnati Knee Rating Score, IKDC Score) verglichen mit präoperativen Werten. Der MOCART Score erreichte zum letzten Untersuchungszeitpunkt 62,4 ± 18,9 Punkte, wobei sich die subchondrale Lamelle nur in 1 Fall (4,3 %) vollständig rekonstruiert darstellte und in 95,7 % nicht intakt war.

Vijayan et al. publizierten 2012 die Ergebnisse von 14 Patienten mit osteochondralen Defekten mit einer durchschnittlichen Defektgröße von 7,2 cm^2^ und einem Durchschnittsalter von 23,6 Jahren. Dabei wurde die ossäre Komponente mit Spongiosa aus den Femurkondylen aufgefüllt und die Knorpelzelltransplantation mit 2 Membranen in Sandwichtechnik durchgeführt. Die Patienten zeigten eine Verbesserung des Cincinnati Knee Scores von 45,1 auf 82,8, und der VAS verbesserte sich signifikant von 7,3 auf 1,7.

## Abkürzungen

ACI: Autologe Chondrozytenimplantation
ACT: Autologe Chondrozytentransplantation
AMIC: Autologous matrix-induced chondrogenesis
AOT: Autologe osteochondrale Transplantation
CPM: Continuous passive motion
CT: Computertomographie
ICRS: International Cartilage Regeneration & Joint Preservation Society
IKDC: International Knee Documentation Committee
MACT: Matrixgestützte autologe Chondrozytentransplantation
MOCART: Magnetic Resonance Observation of Cartilage Repair Tissue
MRT: Magnetresonanztomographie
OATS: Osteochondral autograft transplant system
OCD: Osteochondritis dissecans
